# Emerging trends and hot spots in hypothyroidism research from 2010 to 2025: A bibliometric analysis

**DOI:** 10.1097/MD.0000000000044259

**Published:** 2026-03-06

**Authors:** Zongliang Yu, Anran Gao, Qiaoning Yang, Rui Gao

**Affiliations:** a Beijing University of Chinese Medicine, Beijing, China; b Xiyuan Hospital, China Academy of Chinese Medical Sciences, Beijing, China.

**Keywords:** bibliometric analysis, future research, global trends, hot spots, hypothyroidism

## Abstract

**Background:**

Research in hypothyroidism has expanded significantly in the last decade, resulting in a vast array of studies that pose challenges in comprehensive review and assessment. Bibliometric methodologies serve as quantitative tools to analyze and understand the current research progress and focal areas within this field.

**Methods:**

A systematic search was conducted for articles and reviews pertaining to hypothyroidism within the Core Collection database of Web of Science from 2010 to 2024. Utilizing bibliometric software, notably visualization of similarities viewer and CiteSpace, we performed analyses to assess the contributions, co-occurrence relationships among various references, countries/regions, institutions, journals, and keywords associated with hypothyroidism research.

**Results:**

This study encompassed 16,274 English-language papers published across 2504 journals, originating from 10,616 institutions in 137 countries/regions. Notably, the quantity of hypothyroidism-related publications has exhibited a consistent upward trajectory over the past decade, underscored by robust international collaboration. The United States emerged as the leading contributor in terms of publication count and demonstrated a high concentration of prolific research institutions. The journal “THYROID” prominently featured as the primary publication source for hypothyroidism research, having disseminated several pivotal treatment guidelines within this domain. Recent research has notably spotlighted specific subsets, such as hypothyroidism during pregnancy. Moreover, recent trends have steered focus towards metabolic ailments associated with hypothyroidism, encompassing diabetes, obesity, and exploring the role of dietary supplements.

**Conclusion:**

Leveraging bibliometric tools enables an objective and comprehensive analysis of pertinent data, shedding light on the evolving dynamics within the field of hypothyroidism research.

## 1. Introduction

Hypothyroidism, characterized by generalized hypometabolism due to reduced synthesis or secretion of thyroid hormones or thyroid hormone resistance,^[1]^ predominantly stems from Hashimoto thyroiditis in adults. However, other autoimmune conditions, radiation therapy, or thyroidectomy stand as significant risk factors for hypothyroidism.^[2,3]^ In iodine-sufficient regions, its prevalence spans 1% to 2%, exhibiting a 10-fold higher occurrence in women than men and notably impacting maternal health during pregnancy.^[4–6]^

The early stages of untreated hypothyroidism often lack specific manifestations, making its detection challenging. In severe instances, it may culminate in heart failure and respiratory issues, profoundly compromising patients’ health and quality of life.^[7–9]^ While L-thyroxine remains the cornerstone treatment, it appears insufficient in rectifying the array of metabolic abnormalities stemming from prolonged hypothyroidism.^[10,11]^ There is burgeoning research focusing on novel drug interventions targeting micronutrient supplementation and mitigating comorbidities.^[12,13]^

Over the past decade, the proliferation of over 10,000 hypothyroidism-related studies has prompted the application of new literature review methods for systematic evaluation within specific domains. Widely employed software like CiteSpace and visualization of similarities viewer (VOSviewer) utilize statistical methodologies to quantitatively assess scientific literature, enabling the analysis of data distribution, characteristics, and patterns from diverse perspectives.^[14]^ This study aims to employ bibliometric methods to explore current cutting-edge issues and scientific advancements in hypothyroidism from 2010 to 2025. By doing so, researchers can gain a comprehensive understanding of prevailing research trends in this domain.

## 2. Materials and methods

### 2.1. Data sources and search strategies

The data for this study were sourced from the Core Collection database of Web of Science. Renowned for its comprehensive coverage and systematic indexing, this database is widely accepted for scientometric analysis of scientific literature.^[15–17]^ Searches conducted on June 20, 2025, aimed to minimize bias arising from daily database updates. The search strings used were as follows: “TS = (‘Hypothyroidism’) OR TI = (‘Hypothyroidism*’) OR TI = (Thyroid Stimulating Hormone Deficienc*) OR TI = (‘TSH Deficienc*’).” The search was confined to English-language articles and reviews published between January 2010 and June 2025, initially screened based on titles and abstracts, resulting in 17,393 relevant articles on hypothyroidism.

### 2.2. Data processing

Utilizing the Web of Science database, we extracted information pertaining to annual paper distribution, geographical distribution, affiliations, and citation counts. Full Record and Cited References information for the selected documents was exported in plain text format. These documents were meticulously organized and visualized through CiteSpace.

### 2.3. Bibliometric analysis

CiteSpace, a statistical analysis program by Professor Chao-Mei Chen, was employed to analyze the status and trends of scientific literature.^[18,19]^ Configured at version 5.7 R5, our analysis spanned from 2010 to 2025, utilizing a yearly time slice and selecting the top 50 for analysis in each time frame. Clustering labels were derived using keywords to extract noun terms. VOSviewer (version 1.6.18; Leiden University, Leiden, Netherlands) was also employed to visualize collaborative interactions between countries, institutions, journals, and high-frequency keywords. Notably, node sizes in VOSviewer are indicative of their frequency of appearance in both titles and abstracts.^[20]^ This study complies with the requirements of the BIBLIO checklist.^[21]^

## 3. Results

### 3.1. Annual growth trend

**Figure 1.**
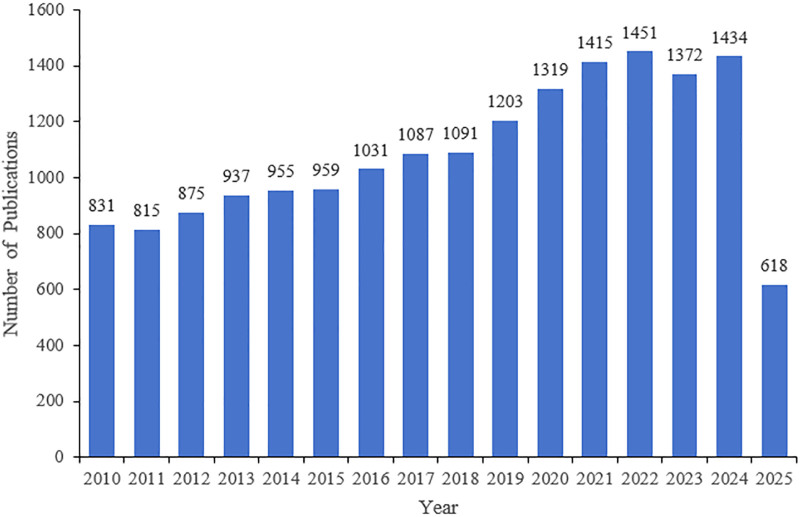
Trends in the number of publications concerning hypothyroidism.

The comprehensive screening of data in the Core Collection database of Web of Science from 2010 to 2025 revealed 17,393 studies related to hypothyroidism, boasting an average citation count of 22.20. Among these, 14,954 (85.98%) comprised original articles, while 2439 (14.02%) were reviews. The trend in article publications is depicted in Figure 1, showcasing a progressive rise from 831 articles in 2010 to 1434 in 2024.

### 3.2. Analysis of co-cited references: clusters and citation burst of research

**Figure 2.**
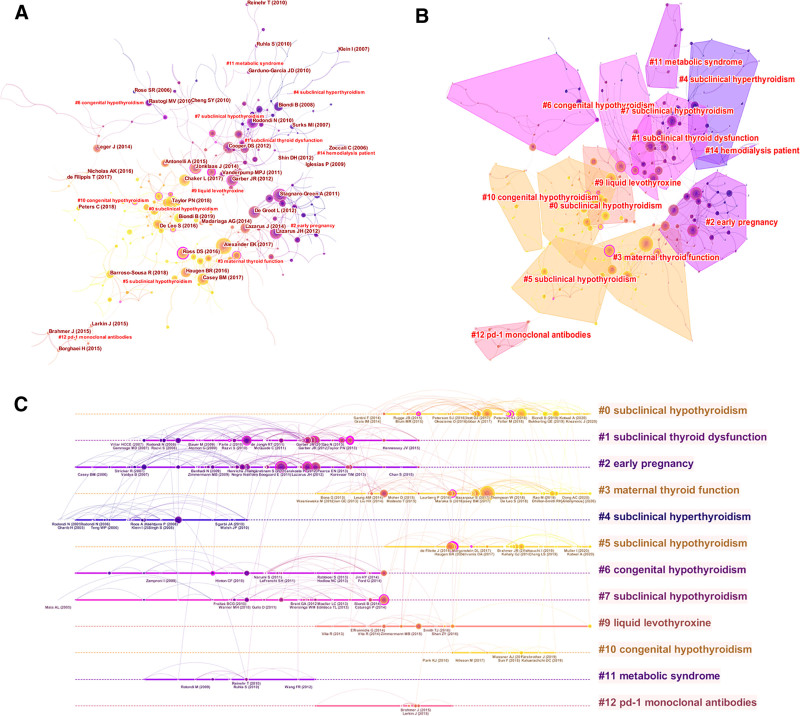
(A) Knowledge map of references related to hypothyroidism research. (B) The co-citation clusters of references related to hypothyroidism research. (C) The timelineview of co-citation clusters.

Co-citation, where 2 publications are linked through a 3rd citation, serves as a repository of domain knowledge, with higher citation frequencies denoting greater importance in the field.^[22]^ Employing Citespace software (Drexel University, Philadelphia) generated a co-occurrence map illustrating the co-cited literature network (Fig. 2A).

Our analysis, displayed in Table 1, highlights the top 10 most co-cited references, comprising 6 articles and 4 reviews. Notably, 7 of these pivotal papers originated from the United States, while 1 each hailed from Netherlands, Wales, and Switzerland. Among these references, Alexander EK’s paper “2017 Guidelines of the American Thyroid Association for the Diagnosis and Management of Thyroid Disease During Pregnancy and the Postpartum” in THYROID was the most co-cited (n = 542), marking its significance in the field. It is followed by “Global epidemiology of hyperthyroidism and hypothyroidism,” which was published in the journal NATURE REVIEWS ENDOCRINOLOGY (n = 321).

**Table 1:** Top 10 co-cited references in the research of hypothyroidism.

Publication year	First author	Country/region	Title	Type	Journal	Citation	Centrality	IF (2024)
2017	Alexander EK^[23]^	USA	2017 Guidelines of the American Thyroid Association for the Diagnosis and Management of Thyroid Disease During Pregnancy and the Postpartum	Article	THYROID	542	0.23	6.7
2018	Taylor PN^[4]^	Wales	Global epidemiology of hyperthyroidism and hypothyroidism	Review	NAT REV ENDOCRINOL	321	0.02	40.0
2017	Chaker L^[1]^	Netherlands	Hypothyroidism	Review	LANCET	312	0.06	88.5
2019	Biondi B^[24]^	USA	Subclinical Hypothyroidism A Review	Review	JAMA-J AM MED ASSOC	305	0.05	55.0
2011	Stagnaro-Green A^[25]^	USA	Guidelines of the American Thyroid Association for the Diagnosis and Management of Thyroid Disease During Pregnancy and Postpartum	Article	THYROID	259	0.10	6.7
2014	Jonklaas J^[26]^	USA	Guidelines for the Treatment of Hypothyroidism: Prepared by the American Thyroid Association Task Force on Thyroid Hormone Replacement	Article	THYROID	258	0.17	6.7
2012	De Groot L^[27]^	USA	Management of Thyroid Dysfunction during Pregnancy and Postpartum: An Endocrine Society Clinical Practice Guideline	Article	J CLIN ENDOCR METAB	237	0.16	5.1
2010	Rodondi N^[28]^	Switzerland	Subclinical Hypothyroidism and the Risk of Coronary Heart Disease and Mortality	Review	JAMA-J AM MED ASSOC	192	0.13	55.0
2012	Cooper DS^[29]^	USA	Subclinical thyroid disease	Article	LANCET	180	0.04	88.5
2017	Casey BM^[30]^	USA	Treatment of Subclinical Hypothyroidism or Hypothyroxinemia in Pregnancy	Article	NEW ENGL J MED	178	0.12	78.5

IF: impact factor, USA = The United States of America.

Cluster analysis delineated 39 clusters, with 12 clusters showcasing more than 10 nodes, as depicted in Figure 2B. A *Q* value exceeding 0.3 and an *S* value surpassing 0.5 signify significant and reasonable clustering outcomes, respectively (*Q* = 0.7787; *S* = 0.928), indicating the stability of our analysis. A timeline view (Fig. 2C) portrays clustering patterns from 2010 to 2025. Noteworthy clusters include “subclinical hyperthyroidism,” “metabolic syndrome,” “congenital hypothyroidism,” and others, each reflecting evolving scientific inquiries based on titles, abstracts, and summaries.

The phenomenon of citation bursts, indicating sudden spikes in citations for specific literature, emphasizes their heightened scholarly focus. Table 2 highlights the top 20 references with the most intense citation bursts. The “clinical significance of subclinical thyroid dysfunction,” along with related treatment guidelines from the THYROID, received significant attention, especially regarding subpopulation studies such as pregnancy and postpartum.

**Table 2:**
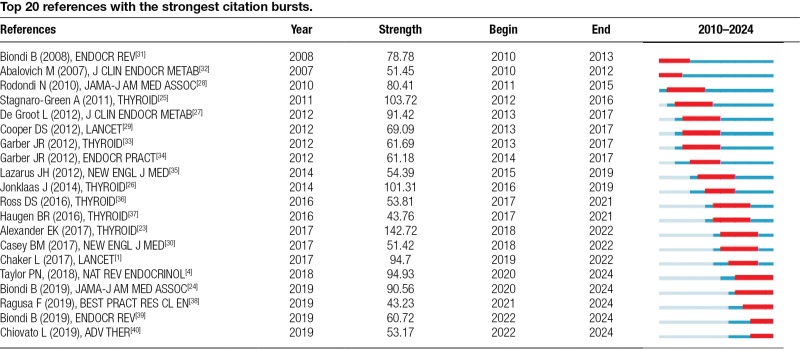
Top 20 references with the strongest citation bursts.

In the last 5 years, a noticeable shift towards epidemiologic studies and evidence-based medical research is observed. Particularly explosive in citations is the global epidemiology of hyperthyroidism and hypothyroidism published in the NAT REV ENDOCRINOL. Additionally, articles like “Subclinical Hypothyroidism: A Review” was featured in JAMA, garnered substantial scholarly attention. This trend underscores a sustained advancement in hypothyroidism research, evident in annual citation surges that persisted until 2025.

### 3.3. Analysis of country/region and institutions

Between 2010 and 2025, hypothyroidism research involved 143 countries and regions. Notably, the United States, China, and Italy accounted for almost half of the total publications, emerging as the top 3 contributors. With 3703 publications, 129,970 citations, and 47 highly cited papers, the United States demonstrated its leadership in hypothyroidism research, as depicted in Table 3. Scrutinizing the average citation numbers, the Netherlands, the United Kingdom, and Germany showcased commendable article quality, displaying excellence in both citation per article and the number of highly cited papers. While China ranked 2nd in the number of articles published, the citation rate reflected room for improvement, a similar trend observed in Turkey and India.

**Table 3:** Top 10 productive countries ranked by publications.

Rank	Country/region	Count	Citation	Citation per article	Highly cited papers	
					Count	Proportion
1	USA	3703	129,970	35.10	47	1.27
2	China	2967	39,429	13.29	12	0.40
3	Italy	1561	49,296	31.58	18	1.15
4	UK	910	40,719	44.75	16	1.76
5	Japan	880	20,167	22.92	8	0.91
6	Turkey	783	10,678	13.64	1	0.13
7	Germany	761	25,743	33.83	11	1.45
8	Netherlands	684	35,125	51.35	18	2.63
9	Brazil	665	16,260	24.45	5	0.75
10	India	607	7978	13.14	2	0.33

**Figure 3.**
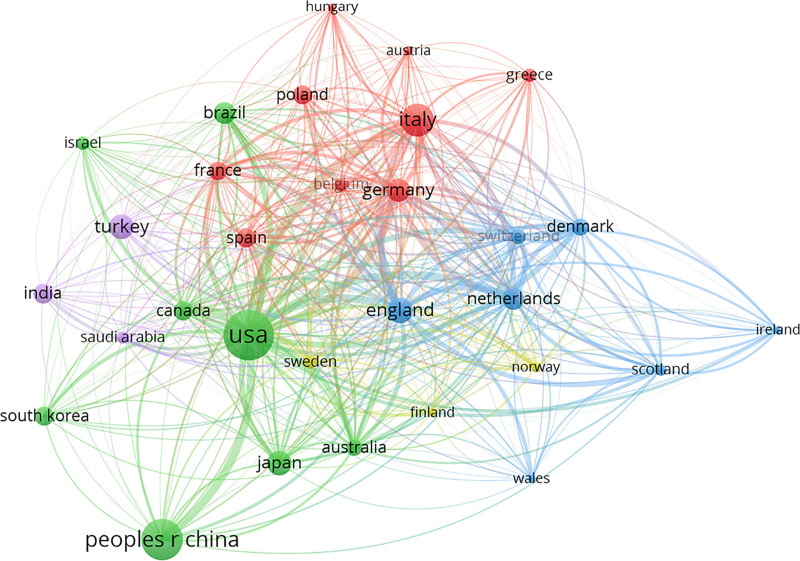
The cross-country/region collaboration visualization map engaged in hypothyroidism. The lines between nodes represent cooperation between countries. Each node represents a country or region. The size of the node is proportional to the number of documents published.

Figure 3 visually depicts source countries/regions, where node size denotes the number of published articles, while lines connecting nodes illustrate the strength of inter-country cooperation. Among the top 10 countries in article publications, the United States, England, and the Netherlands exhibited the highest total link strength, suggesting closer collaborations. Conversely, although India and Turkey contributed more publications, limited inter-country cooperation signifies potential research disparities in developing countries.

Of the 12,077 research institutions involved in hypothyroidism studies, the top 10 institutions contributed 2606 articles. As depicted in Table 4, institutions with substantial publications were concentrated in the United States (3 institutions) and the Netherlands (2 institutions). According to citation counts, the institutions in descending order were Harvard University (24,616), University of California system (24,094), and National Institutes of Health (16,133), affirming the research stature of the United States in hypothyroidism, corroborated by the average citation per article.

**Table 4:** Top 10 productive institutions ranked by publications.

Rank	Institution	Count	Citation	Citation per article	Highly cited papers	
					Count	Proportion
1	Harvard university	416	24,616	59.17	16	3.85
2	University of California system	357	24,094	67.49	11	3.08
3	University of London	259	14,340	55.37	7	2.70
4	National institutes of health, USA	254	16,133	63.52	9	3.54
5	Institut national de la sante et de la recherche medicale inserm	238	9910	41.64	7	2.94
6	Erasmus University Rotterdam	235	14,097	59.99	9	3.83
7	Egyptian Knowledge Bank	230	3698	16.08	0	0.00
8	University of Copenhagen	214	7754	36.23	4	1.87
9	University of Amsterdam	203	9582	47.20	5	2.46
10	University of Pisa	200	7169	35.85	2	1.00

### 3.4. Analysis of journals and co-cited journals

All papers spanned 2645 academic journals. Among the top 10 journals based on the number of papers published (Table 5), THYROID led with 586 publications, boasting an impact factor (IF) of 6.7 in 2024. Subsequent journals included JOURNAL OF CLINICAL ENDOCRINOLOGY & METABOLISM, FRONTIERS IN ENDOCRINOLOGY, CLINICAL ENDOCRINOLOGY, and ENDOCRINE. Five of the top 10 journals belonged to the *Q*1 division in Journal Citation Reports, with THYROID having the highest impact factor at 6.7.

**Table 5:** Top 10 journals ranked by number of publications.

Rank	Journal	Documents	Country	IF	JCR (2024)
1	THYROID	586	USA	6.7	Q1
2	JOURNAL OF CLINICAL ENDOCRINOLOGY & METABOLISM	546	USA	5.1	Q1
3	FRONTIERS IN ENDOCRINOLOGY	540	Switzerland	4.6	Q1
4	CLINICAL ENDOCRINOLOGY	280	UK	2.4	Q3
5	ENDOCRINE	271	USA	2.9	Q3
6	JOURNAL OF PEDIATRIC ENDOCRINOLOGY & METABOLISM	248	UK	1.0	Q4
7	PLOS ONE	224	USA	2.6	Q2
8	EUROPEAN JOURNAL OF ENDOCRINOLOGY	218	UK	5.2	Q1
9	JOURNAL OF ENDOCRINOLOGICAL INVESTIGATION	201	Italy	3.5	Q2
10	SCIENTIFIC REPORTS	181	UK	3.9	Q1

IF = impact factor, JCR = Journal Citation Reports, UK = The United Kingdom of Great Britain and Northern Ireland, USA = The United States of America.

Influence of a journal hinges on its citation count, signifying its intrinsic impact in a particular field. Table 6 reveals the most frequently cited journals, led by JOURNAL OF CLINICAL ENDOCRINOLOGY & METABOLISM (n = 10,319), THYROID (n = 8681), CLINICAL ENDOCRINOLOGY (n = 6586), and EUROPEAN JOURNAL OF ENDOCRINOLOGY (n = 6310). These journals, part of the most published list, belong to 7 Q1 journals. Notably, 7 of the top 10 journals hailed from the United States, while the remaining 3 originated from the United Kingdom.

**Table 6:** Top 10 co-cited journals ranked by number of citations.

Rank	Co-cited journal	Citations	Country	IF	JCR (2024)
1	JOURNAL OF CLINICAL ENDOCRINOLOGY & METABOLISM	10,319	USA	5.1	Q1
2	THYROID	8681	USA	6.7	Q1
3	CLINICAL ENDOCRINOLOGY	6586	UK	2.4	Q3
4	EUROPEAN JOURNAL OF ENDOCRINOLOGY	6310	UK	5.2	Q1
5	NEW ENGLAND JOURNAL OF MEDICINE	6107	USA	78.5	Q1
6	LANCET	4316	UK	88.5	Q1
7	ENDOCRINE REVIEWS	3766	USA	22.0	Q1
8	PLOS ONE	3540	USA	2.6	Q2
9	JAMA-JOURNAL OF THE AMERICAN MEDICAL ASSOCIATION	3459	USA	55.0	Q1
10	ENDOCRINOLOGY	3446	USA	3.3	Q2

IF = impact factor, JCR = Journal Citation Reports, UK = The United Kingdom of Great Britain and Northern Ireland, USA = The United States of America.

### 3.5. Hotspots and frontiers analysis

**Figure 4.**
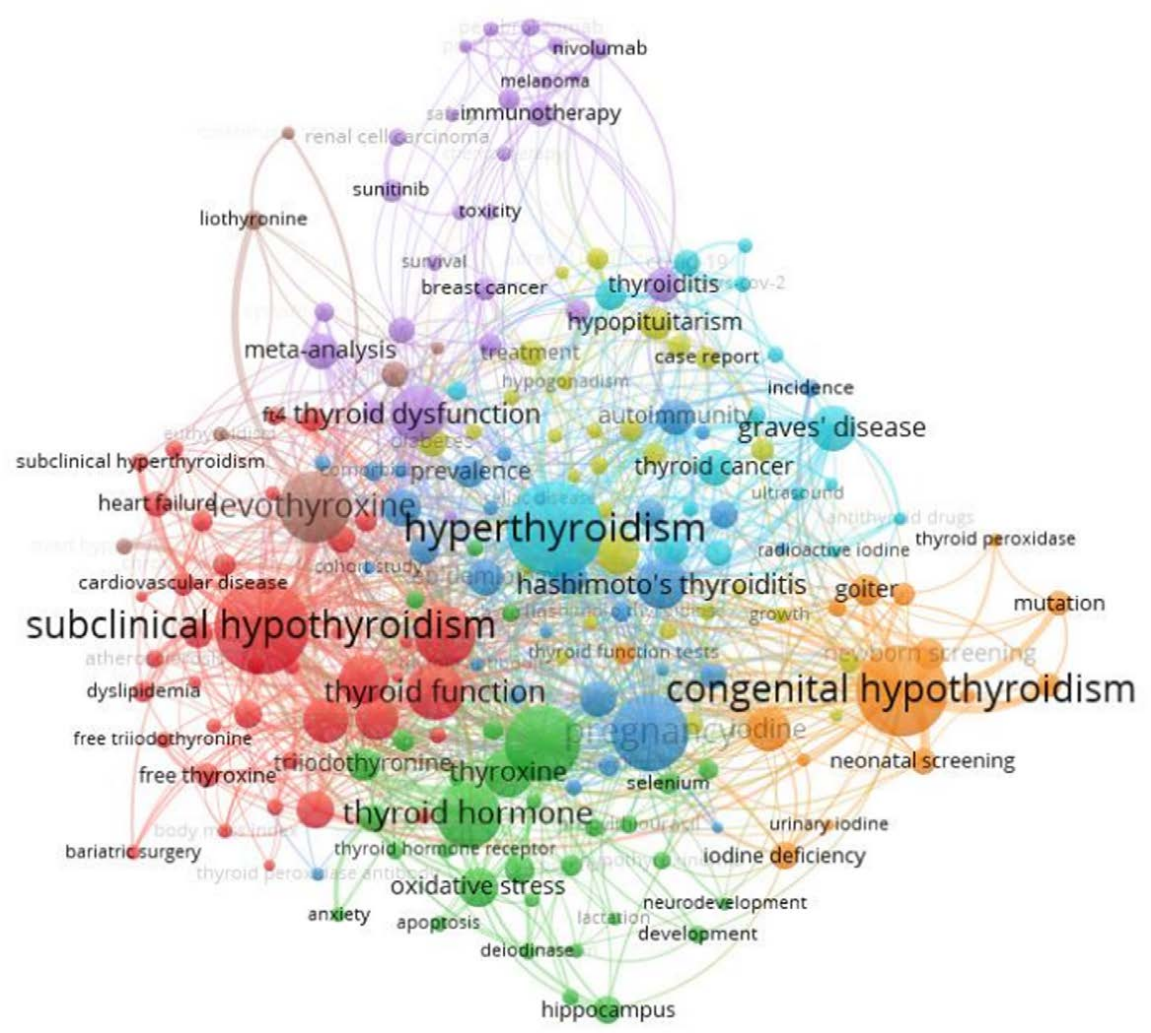
Co-occurrence network of keywords in the field of hypothyroidism.

We used VOSviewer to construct a network graph (Fig. 4) of keywords that have been widely used in research and represent the main directions of current research. The figure shows 8 clusters, each represented by a different color. Meaningless words such as hypothyroidism and thyriod were excluded. The keywords that appeared more frequently are shown in Table 7, mainly including hyperthyroidism, hashimoto thyroiditis, Graves’ disease, thyroid cancer, and other thyroid disorders that are closely related to the etiology or prognosis of hypothyroidism. Also included are subclinical hypothyroidism, congenital hypothyroidism, pregnancy, newborn screening, and other subgroups of the study population of wide interest in the field of hypothyroidism. The presence of the keywords obesity, iodine, metabolic syndrome, diabetes, and reflects the close association of hypothyroidism with metabolic disorders. The meta-analysis, epidemiology, prevalence, risk factors reflect the application of epidemiologic and evidence-based medical research in the field of hypothyroidism. And the keywords of oxidative stress and rat reflect the dynamics of experimental research in this field. The above keywords basically represent the connotation of their clustering.

**Table 7:** Top 20 keywords in the research of hypothyroidism.

Rank	Keywords	Count	Rank	Keywords	Count
1	Hyperthyroidism	698	11	Oxidative stress	197
2	Subclinical hypothyroidism	681	12	Meta-analysis	266
3	Congenital hypothyroidism	645	13	Thyroid cancer	232
4	Pregnancy	492	14	Epidemiology	227
5	Levothyroxine	438	15	Newborn screening	157
6	TSH	332	16	Prevalence	201
7	Hashimoto thyroiditis	253	17	Rat	102
8	Graves’ disease	219	18	Metabolic syndrome	198
9	Obesity	213	19	Diabetes	199
10	Iodine	203	20	Risk factors	134

CiteSpace was used to screen keywords with strong citation outbreaks, as shown in Table 8. Thyroid-related clinical studies such as carcinoma, metabolism, double blind, autoantibody, etc that started to get citation outbursts in 2010. In 2012, the research hotspot shifted to coronary heart disease, rat, insulin resistance, and adolescent, emphasizing hypothyroidism-related diseases and subpopulation studies. After 2018, it developed into gene expression, oxidative stress, guideline, epidemiology and other mechanism studies, and epidemiological studies. In recent years, more attention has been paid to therapeutic agents for thyroid disorders such as levothyroxine, which remains at the forefront to this day.

**Table 8:** Keywords with the strongest citation bursts.

Keywords	Year	Strength	Begin	End	2010–2024
Carcinoma	2010	38.56	2010	2013	▃▃▃▃▂▂▂▂▂▂▂▂▂▂▂
Metabolism	2010	25.57	2010	2013	▃▃▃▃▂▂▂▂▂▂▂▂▂▂▂
Double blind	2010	44.72	2010	2015	▃▃▃▃▃▃▂▂▂▂▂▂▂▂▂
Autoantibody	2010	42.87	2012	2015	▂▂▃▃▃▃▂▂▂▂▂▂▂▂▂
Coronary heart disease	2010	51.21	2012	2017	▂▂▃▃▃▃▃▃▂▂▂▂▂▂▂
Rat	2010	20.51	2014	2017	▂▂▂▂▃▃▃▃▂▂▂▂▂▂▂
Insulin resistance	2010	26.94	2014	2019	▂▂▂▂▃▃▃▃▃▃▂▂▂▂▂
Adolescent	2010	29.37	2016	2017	▂▂▂▂▂▂▃▃▂▂▂▂▂▂▂
Gene expression	2010	8.96	2018	2019	▂▂▂▂▂▂▂▂▃▃▂▂▂▂▂
Oxidative stress	2010	10.66	2018	2021	▂▂▂▂▂▂▂▂▃▃▃▃▂▂▂
Guideline	2010	51.91	2018	2023	▂▂▂▂▂▂▂▂▃▃▃▃▃▃▂
Epidemiology	2010	40.84	2018	2023	▂▂▂▂▂▂▂▂▃▃▃▃▃▃▂
Receptor	2010	22.6	2020	2021	▂▂▂▂▂▂▂▂▂▂▃▃▂▂▂
Iodine	2010	22.12	2020	2024	▂▂▂▂▂▂▂▂▂▂▃▃▃▃▃
Levothyroxine	2010	11.07	2020	2024	▂▂▂▂▂▂▂▂▂▂▃▃▃▃▃

## 4. Discussion

### 4.1. General information

The ability to obtain up-to-date information and identify research trends and focal points is crucial for clinical practitioners engaging in scientific inquiry. Bibliometric methodologies have recently thrived, enabling swift comprehension of pivotal issues and hotspots within specific research domains.^[14]^ This paper innovatively aims to systematically organize and visualize the research focuses and hotspots in hypothyroidism research using bibliometric tools like CiteSpace and VOSviewer. Such an approach intends to aid clinical workers in conducting more effective scientific investigations.

In this study, a comprehensive literature search in the Web of Science database retrieved 17,393 English-language papers published between 2010 and 2025, encompassing 2645 journals from 12,077 institutions across 143 countries/regions. Notably, there has been a progressive growth in publications concerning hypothyroidism over the past decade. The United States stands out as the leading country in both publications and concentration of prolific research institutions. Its contribution of 47 highly cited papers underscores its leadership in hypothyroidism research. Meanwhile, countries like the Netherlands and the United Kingdom exhibit high-quality articles and substantial international collaborations, depicting extensive global cooperation in this field. Over half of the top journals in the field of internal medicine, classified under the Q1 division, suggest continued widespread interest in hypothyroidism and related disorders. THYROID emerges as the journal publishing the most research in this field, notably featuring the top 3 crucial publications focusing on subpopulation studies such as pregnancy and postpartum.

Based on reference analysis and keyword extraction, research in hypothyroidism has gravitated towards hashimoto thyroiditis, thyroid cancer, and other closely linked thyroid disorders. Subgroups like subclinical hypothyroidism and pregnancy have been prominent focal points. Recent years have seen increased attention toward metabolic disorders associated with hypothyroidism, such as obesity, metabolic syndrome, and diabetes. However, the evolution of new directions like evidence-based medical research, mechanism exploration involving oxidative stress, and drug research such as levothyroxine may decelerate research pace in previous hot areas.

### 4.2. Research hotspots

#### 4.2.1. Metabolic diseases and hypothyroidism

The intimate association between hypothyroidism and metabolic diseases, notably diabetes mellitus, highlights a significant research focus.^[41]^ A substantial cohort study establishes hypothyroidism as a pivotal risk factor for new-onset diabetes mellitus.^[42]^ Furthermore, hypothyroidism disrupts glucose and lipid metabolism, amplifying the risk of insulin resistance, diabetes, and its associated complications.^[43–45]^ Studies also underscore how thyroid dysfunction during pregnancy heightens the likelihood of developing gestational diabetes, extending to an augmented risk of type 2 diabetes in offspring.^[46–48]^ Moreover, patients with type 1 diabetes often grapple with autoimmune thyroid disease and eventually leads to hypothyroidism.^[49–52]^

Exploration into the interplay between hypothyroidism and obesity, as well as lipid metabolism abnormalities, remains a significant research hotspot. Hypothyroidism’s influence on lipid metabolism and its role as a key factor in obesity has garnered attention.^[53,54]^ Studies revealing an elevated prevalence of metabolic syndrome and obesity in individuals with higher thyroid stimulating hormone levels further substantiate this relationship.^[55]^ The impact of hypothyroidism on the risk of metabolic syndrome in women has been further confirmed in a study of a Chinese population.^[56]^ Nevertheless, delineating the causal relationship between obesity and hypothyroidism proves challenging due to the broad associations of metabolic diseases. The bidirectional link between obesity and hypothyroidism underscores the potential impact of obesity prevention on thyroid disorder treatment.^[57,58]^

Notably, in obese patients, high levels of thyroid stimulating hormone can raise serum pro-inflammatory factors and increase the risk of cardiovascular disease.^[59]^ In addition, hypothyroidism is capable of inducing abnormalities in homocysteine metabolism,^[60]^ with potential joint effects with the adverse effects of diabetes mellitus and abnormalities in lipid metabolism on cardiovascular disease,^[61,62]^ the mechanism of which may be related to the induced inflammatory response.^[63]^ Overall, with the cross-fertilization of metabolic and cardiovascular diseases, the link between hypothyroidism and abnormalities of lipid metabolism and atherosclerosis deserves in-depth study.^[62,64]^

#### 4.2.2. Thyroid cancer and hypothyroidism

The relationship between hypothyroidism and thyroid cancer has generated recent controversy.^[65,66]^ Some studies suggest an absence of association between thyroid cancer development and hypothyroidism.^[67]^ Conversely, an analysis of 247,107 electronic health records indicates a strong connection between the thyroid stimulating hormone polygenic score and thyroid cancer development.^[68]^ Moreover, findings propose that Hashimoto thyroiditis, a primary factor in hypothyroidism, might exert a protective effect against thyroid cancer outcomes.^[69–71]^ Further investigation is warranted to elucidate these specific connections.

Notably, hypothyroidism commonly emerges as a complication in postoperative and maintenance therapy for thyroid cancer.^[72]^ Thyroid replacement therapy stands as standard practice post-thyroid cancer treatment,^[73]^ and thyroxine and thyroid stimulating hormone tests serve as pivotal indicators for postoperative thyroid gland observation.^[74,75]^ Notably, free triiodothyronine has been highlighted as the most influential factor impacting the quality of life in patients with differentiated thyroid cancer post-surgery.^[73]^ Studies have identified increased risks of hypothyroidism among patients treated with cervical and craniospinal irradiation regimens compared to systemic irradiation therapy.^[76]^ Additionally, endothelial impairment and abnormal lipid metabolism induced during radioactive iodine therapy for thyroid cancer associate with subsequent hypothyroidism.^[77,78]^

#### 4.2.3. Oxidative stress and hypothyroidism

Numerous experimental studies confirm hypothyroidism’s capacity to induce oxidative stress across various systems. Studies demonstrate oxidative stress and DNA damage in the mammary gland as early occurrences in experimental hypothyroidism. Elevated thyroid stimulating hormone levels are capable of triggering oxidative stress in mammary cells, promoting reactive oxygen species production, and destabilizing the genome.^[79]^ Chronic hypothyroidism, resulting in a reduced ovulation rate, is associated with disrupted antioxidant systems.^[80]^ Moreover, female hypothyroid rats display increased susceptibility to placental restriction and fetal demise due to activated oxidative stress and endoplasmic reticulum stress at the maternal–fetal interface.^[81]^ Nevertheless, ongoing discussions exist regarding whether hypothyroidism-induced oxidative stress directly links to the disease or indirectly results from alterations in the lipid profile.^[82]^ Clinical studies report higher levels of oxidative inflammation and stress among hypothyroid patients,^[83,84]^ associated with heightened expression of oxidative stress-related genes and decreased serum antioxidant effects.^[85]^

#### 4.2.4. Novel treatments for hypothyroidism

Although L-thyroxine remains the classic and effective treatment for hypothyroidism, recent studies have explored the unique effects of other drugs and therapies in managing the condition. Investigating the role of vitamins in hypothyroidism has become a prominent research focus. Randomized controlled trials suggest that combined zinc, magnesium, and vitamin A supplementation reduces serum free thyroxine and high-sensitivity C-reactive protein levels in hypothyroid patients.^[86]^ Vitamin E demonstrates potential in improving brain-derived neurotrophic factor, preventing oxidative brain tissue damage, and ameliorating learning and memory deficits in juvenile hypothyroid rats.^[87]^ Similarly, vitamin D showcases potential in enhancing learning and memory, averting brain tissue damage, and alleviating associated liver dysfunction in hypothyroid rats.^[88,89]^

Oxidative stress induced by hypothyroidism closely associates with atherosclerosis and nonfat metabolic liver disease. Investigations exploring peroxisome proliferator-activated receptor γ agonists, such as pioglitazone and rosiglitazone, suggest their efficacy in reducing oxidative damage in rat heart and aortic tissues induced by hypothyroidism.^[90]^ Administration of Nano Sel demonstrates reduced oxidative stress levels, inhibits cardiac fibrosis and aortic injury in hypothyroid rats, and exerts cardioprotective effects.^[91]^ Additionally, the role of certain natural active ingredients is gaining attention. Thymoquinone exhibits the potential to upregulate sirtuin 1 expression and limit hypothyroidism-induced structural changes in the testes, indicating a potential supplementary role in hypothyroidism management.^[92]^ Quercetin regulates platelet aggregation in hypothyroid rats, thereby preventing thrombosis.^[93]^ Similarly, hesperidin modulates inflammatory pathways and attenuates hypothyroidism-induced lung injury in rats.^[94]^

## 5. Conclusion

In this study, we conducted an analysis of the trends and hotspots in hypothyroidism research over the past decade using bibliometric software like CiteSpace and VOSviewer. The findings highlighted an overall increasing trend in hypothyroidism-related publications with extensive international collaborations. The focus in recent years encompassed pregnancy and other population subgroups, metabolic diseases related to hypothyroidism, oxidative stress mechanisms, and novel therapeutic research. Unlike traditional reviews, analyses based on bibliometric tools provide a clearer illustration of evolving research priorities and trends, offering relatively objective data analysis.

## Author contributions

**Conceptualization:** Zongliang Yu.

**Data curation:** Anran Gao, Qiaoning Yang.

**Formal analysis:** Zongliang Yu, Anran Gao.

**Funding acquisition:** Rui Gao.

**Investigation:** Rui Gao.

**Methodology:** Zongliang Yu.

**Project administration:** Rui Gao.

**Software:** Anran Gao.

**Supervision:** Rui Gao.

**Visualization:** Qiaoning Yang.

**Writing – original draft:** Zongliang Yu, Anran Gao.

**Writing – review & editing:** Qiaoning Yang, Rui Gao.
